# Duration of Untreated Psychosis in Chinese and Mauritian: Impact of Clinical Characteristics and Patients’ and Families’ Perspectives on Psychosis

**DOI:** 10.1371/journal.pone.0157083

**Published:** 2016-06-09

**Authors:** Jaya Prishni Devi Thakoor, Huixi Dong, Xiaojie Zhang, Gang Wang, Hui Huang, Yutao Xiang, Wei Hao

**Affiliations:** 1 Sir Seewoosagur Ramgoolam National Hospital, Pamplemousses, Mauritius; 2 Mental Health Institute, Second Xiangya Hospital, Central South University, Changsha, China; 3 Brain Research Center, University of British Columbia, Vancouver, Canada; 4 Unit of Psychiatry, Faculty of Health Sciences, University of Macau, Macao SAR, China; Yale University School of Medicine, UNITED STATES

## Abstract

**Background:**

Duration of untreated psychosis (DUP) is a potentially modifiable prognostic factor of course and prognosis of psychiatric disorders. Few studies have demonstrated that different cultural backgrounds or perspectives on psychosis may be important factors to the DUP. This study attempted to explore whether the DUP was different in Chinese and Mauritians and to clarify potential influencing factors to a long DUP (>3 months).

**Methods:**

200 patients from China and 100 patients from Mauritius were enrolled in the study. Their respective family members were also recruited. Demographic and clinical characteristics were collected, and the Internalized Stigma of Mental Illness (ISMI) scale was adapted to measure the stigma in all subjects. Binary logistic regression analysis was used to find the potential influencing factors to the long DUP.

**Results:**

35.3% of the enrolled patients had a long DUP. No significant difference was found in frequency of long DUP between the two countries. Chinese patients had relatively less perceptions of stigma. Furthermore, Chinese patients with a long DUP had more perception of breakup due to mental illness (OR = 2.22, p = 0.04) and more families’ perception of the patient being disinherited due to mental illness (OR = 6.47, p = 0.01). Mauritian patients with a long DUP were less likely to have high monthly income (OR = 0.12, p<0.01), while they had less patients’ awareness of mental illness (OR = 0.31, p<0.05) and less families’ awareness of mental illness (OR = 0.14, p<0.01).

**Conclusion:**

The results of this study underlined the importance of DUP in economic conditions, racial and sociocultural factors, and public awareness on psychosis in developing countries.

## Introduction

The duration of untreated psychosis (DUP), defined as the time from the onset of psychotic symptoms till the start of pharmacological treatment[1], has been gaining more attention in both clinical practice and research over the last two decades. There is not sufficient evidence to draw the conclusion that DUP is a predictor of disease outcome, however it can be a potentially modifiable prognostic factor or a marker of poor course and prognosis of psychiatric disorders. It has been suggested that a DUP of more than 3 months (long DUP) may predict unfavorable prognosis of patients with psychosis [2–3]. For instance, meta-analyses and reviews bearing on schizophrenia have found long DUP correlating significantly with greater positive and negative symptoms severity, less likelihood of remission, poor general symptomatic, social functioning and global outcome [4], and shorter DUP bringing greater response to antipsychotic treatment [5]. Moreover, an extended DUP in bipolar disorders is associated with more mood episodes, more suicidal behavior and a trend towards greater lifetime mood instability [6–7]. These show the importance of approaching a psychiatrist early. Therefore, it is vitally important to identify the factors leading to a long DUP, which may give us the opportunity to identify weaknesses in help-seeking behavior and thus shorten DUP.

Some barriers to the access of mental health services at the level of the patient affect DUP in underdeveloped countries. First, geographic barriers, such as long distance and inconvenient transportation, can prevent the patients from approaching a mental health care facility [8–11]. Second, economic factors may impair access to mental health services. For a low-income population, it may simply be too expensive to afford psychiatric treatment [9–10, 12–13]. Third, the fear of stigmatization and discrimination associated with psychosis can prevent patients, or their family members, from acknowledging the presence of mental disorder and therefore seeking help [8–10, 14–15]. In addition, patients and their families may have various perspectives and attitudes towards different types of mental disorders and subsequently, influence their approach to mental health services.

To our knowledge, much of the research concerning DUP has focused on one site only [2, 12, 16], without direct comparison in different cultures and religions. In addition, previous studies focused on patients’ [8–9, 11, 14, 17–20] or caretakers’ perspectives [21–22], but research on DUP with diverse population or perspectives from both patients and their family members are especially scarce. In this research, both respects were taken into consideration, and the above barriers were assessed in Changsha, China and Mauritius, which are geographically, socially and culturally different. The authors believe this research will yield findings of impact factors for DUP in different backgrounds that can be used to ameliorate mental health care system delivery in both sites.

## Material and Methods

### Study Settings and Participants

This joint study was carried out in the Mental Health Institute of Second Xiangya Hospital (MHI-SXH), Changsha, China and the Brown Sequard Mental Health Care Centre (BSMHCC), Beau Bassin, Mauritius between December, 2011 and January 2013. The MHI-SXH has 150 psychiatric beds with approximately 300 outpatient visits per day and provides psychiatric service for the population of 6.4 million in Changsha—the capital city of Hunan province. The BSMHCC is the only psychiatric hospital in Mauritius with approximately 340 outpatient visits per day and 700 psychiatric beds, and is responsible for the whole Mauritian population (approximately 1.2 million).

Patients and their family members were consecutively approached and invited to participate in the study if they fulfilled the following inclusion criteria: (1) in- or out-patients with a history of psychotic symptoms were reviewed by medical records; (2) age 10 years or above; (3) being Mandarin speaker in China and Creole speaker in Mauritius; (3) being able to understand the contents of the interview and (4) be willing to sign the written informed consent. We obtained the written informed consent from both patients and their families before the interview. Thirty-one patients (10.3%) were under 18 years old and their parents or their legal guardians signed the written informed consent. Patients who did not want to participate or were not accompanied by their family were excluded. The research protocol including the consent procedures was approved by the Ethics committee of the Human Ethics Committee of the Second Xiangya Hospital of Central South University and the National Ethics Committee of the Mauritian Government.

### Instruments and Assessments

Patients’ basic demographic and clinical characteristics were collected by a form designed for this study and then confirmed with their family members. The Structured Clinical Interview for DSM-IV-TR Axis I Disorders-Patient Edition (SCID-I/P), English and Chinese version [23–24] and the Structured Clinical Interview for DSM-IV-TR Axis II Disorders (SCID-II), English and Chinese version [25–26] were administered. Face-to-face interviews were conducted by three trained psychiatrists: one Mauritian and two Chinese, who were masters or doctoral level researchers. Before our survey, all interviews were trained with high inter-rater reliability by the supervision of the corresponding author. We recruited subjects with the current and primary DSM-IV-TR Axis–I diagnoses at the time of admission. Participants who met diagnoses of substance use disorders (except for nicotine use disorder) and personality disorders, who had chronic physical illness and disability, who were incapable of effective communication, and who refused to sign the informed consent were excluded. DSM-IV-TR diagnoses were collapsed into three groups: schizophrenia, major depression, bipolar disorders (The principal diagnosis was used if the patient had more than one diagnosis). The onset date of psychotic symptoms and the date of first consultation with a psychiatrist was retrieved from medical records and confirmed with patients’ families. The criteria of onset of treatment was defined as the initiation of an adequate dose of antipsychotic medication (as recorded in healthcare records), which contained the following points: adhering to dosage levels recommended by the psychiatrist, which continued for a period of at least 1 month, or which led to significant reduction in symptoms as measured by SCID-I/P.

Stigma and discrimination of patients and their families were measured by the Chinese and English versions of a questionnaire design based on the Internalized Stigma of Mental Illness (ISMI) Scale [27]. The ISMI has been broadly used in different countries [28]. It consists of 29 items and may be collapsed into two domains: social and family exclusion; discomfort of significant others.

### Statistical Analysis

The statistical analysis was performed using the Statistical Package for the Social Sciences (SPSS), version 20.0 (SPSS Inc., Chicago, IL, USA). The measurement data were described with mean ± SD and enumeration data were expressed as frequencies (N) and percentage values (%). For the purpose of the study, the samples were respectively divided into two groups: subjects from Changsha and Mauritius or subjects with short DUP (≤3 months) and long DUP (> 3 months). Comparisons between subjects in Changsha and Mauritius or between short and long DUP in terms of demographic and clinical variables and patients’ and families’ perception of stigma were conducted by chi-square tests, Fisher's exact test, or independent t-tests as appropriate. There is only one patient with major depression in “long DUP” in Mauritius. So in order to facilitate statistics, we combine “bipolar disorders” and “major depression” as “mood disorders” in comparison of diagnoses between patients with long and short DUP in Changsha and Mauritius (in comparison of basic demographic and clinical characteristics, and stigma aspects between long and short DUP in Changsha and Mauritius). Furthermore, patients’ long DUP in the two regions were compared after controlling for the potentially confounding effects of variables that significantly differed in univariate analyses using binary logistic regression analysis with the “Enter” method to determine the independent variables significantly associated with long DUP. Statistical significance was set at P value < 0.05 (two-tailed). Furthermore, we calculated post hoc power of all the comparison with no significant differences (except for general demographic variables) when setting α err prob = 0.05 and listed them in results.

## Results

A total of 320 patients met study entry criteria and were invited to participate in the study (210 in MHI-SXH and 110 in BSMHCC). 300 of them finally completed the interview, resulting in a participation rate of 93.8%. Of the patients, 106 (35.3%) had long DUP: 69 (34.5%) in Changsha and 37 (37.0%) in Mauritius. DUP data was presented in Table 1.

**Table 1:** Duration of untreated psychosis (DUP) length for patients in Changsha and Mauritius.

DUP	Changsha (n = 200)		Mauritius (n = 100)	
	N	%	N	%
**<3 months**	131	65.5	63	63.0
**3–11 months**	29	14.5	11	11.0
**1–5 years**	34	17.0	19	19.0
**6–10 years**	5	2.5	3	3.0
**11–15 years**	0	0	2	2.0
**More than 20 years**	1	0.5	2	2.0

Tables 2 and 3 show the socio-demographic, DUP, clinical characteristics and patients’ and their families’ perception of stigma in Changsha and Mauritius. Compared to those in Mauritius, Chinese patients were younger (China: 24.8±8.1 years, vs Mauritius: 40.0±12.8 years t = 12.3, p<0.0001). (Table 2). There was no significant difference in frequency of long DUP between the two sites before (*χ*^*2*^: 0.1, df = 1, p = 0.66, power = 0.07) and after potential confounders were controlled for (F = 2.4, p = 0.11).

**Table 2:** Basic demographic and clinical characteristics of the patients in Changsha and Mauritius.

	Changsha(n = 200)		Mauritius(n = 100)	
	N	%	N	%
**Male sex**	84	42.0	42	42.0
**Married**	51	25.5	41	41.0
**Inpatient**	81	40.5	46	46.0
**Urban residence**	87	43.5	51	51.0
**Diagnosis**				
**Schizophrenia**	149	74.5	74	74.0
**Bipolar disorders**	33	16.5	16	16.0
**Major depression**	18	9.0	10	10.0
**Low education (Junior middle school or below)**	72	36.0	87	87.0
**High monthly income (over RMB 1000)**	154	77.0	33	33.0
**Having insurance**	159	79.5	5	5.0
**Long duration of untreated psychosis(> 3 months)**	69	34.5	37	37.0

^a^ DUP = duration of untreated psychosis.

**Table 3:** Stigma aspects of patients and their families between Changsha and Mauritius.

	Patients’ stigma							Families’ stigma						
	Changsha(n = 200)		Mauritius(n = 100)		Statistics			Changsha (n = 200)		Mauritius(n = 100)		Statistics		
	N	%	N	%	χ^2^	p	power	N	%	N	%	χ^2^	p	power
**Awareness of mental disorder**	160	80.0	72	72.0	2.43	0.12	0.37	126	63.0	64	64.0	0.03	0.87	0.09
**Perception of being laughed at**	56	28.0	45	45.0	8.63	<0.01		61	30.5	34	34.0	0.38	0.54	0.09
**Perception of being avoided**	51	25.5	41	41.0	7.53	<0.01		57	28.5	41	41.0	4.74	0.03	
**Perception of being labeled as ‘mad’**	79	39.5	55	55.0	6.48	0.01		55	27.5	54	54.0	20.24	<0.01	
**Perception of being disinherited due to mental illness**	23	11.5	11	11.0	0.02	0.86	0.05	14	7.0	9	9.0	0.38	0.54	0.09
**Perception of breakup due to mental illness**	43	21.5	24	24.0	0.24	0.62	0.08	43	21.5	24	24.0	0.24	0.62	0.08
**Perception of family being embarrassed because of mental illness**	43	21.5	34	34.0	5.46	0.02		44	22.0	36	36.0	6.68	0.01	
**Perception of loss of job due to mental illness**	68	34.0	48	48.0	5.51	0.02		68	34.0	48	48.0	5.51	0.02	
**Perception of being denied promotions due to mental illness**	32	16.0	43	43.0	25.92	<0.01		39	19.5	37	37.0	10.79	<0.01	

In addition, Chinese patients had less perceptions of being laughed at; Chinese patients and their families both had less perceptions of being avoided, being labeled as ‘mad’, family being embarrassed because of mental illness, loss of job due to mental illness and being denied promotions due to mental illness (Table 3).

Table 4 displays the socio-demographic and clinical characteristics and patients’ and their families’ perception of stigma between patients with long and short DUP in Changsha and Mauritius. For Chinese, age did not differ significantly between short and long DUP (short DUP: 24.2±8.2 years vs long DUP: 26.3±7.9 years, t = -0.69, p = 0.93). Compared to short DUP, long DUP was significantly associated less patients’ and families’ perception of being avoided, more perception of breakup due to mental illness and more families’ perception of being disinherited due to mental illness. For Mauritians, age also did not differ significantly between short and long DUP (short DUP: 40.63.5±13.01 years vs long DUP: 38.95±12.7 years, t = -0.63, p = 0.81). Compared to short DUP, long DUP was significantly associated with less high monthly income, less patients’ and families’ awareness of mental disorder, more perception of breakup due to mental illness.

**Table 4:** Comparison of basic demographic and clinical characteristics, and stigma aspects between long and short DUP in Changsha and Mauritius.

	Changsha (n = 200)							Mauritius (n = 100)						
	Short DUP (n = 131)		Long DUP (n = 69)		Statistics			Short DUP(n = 63)		Long DUP(n = 37)		Statistics		
	N	%	N	%	χ^2^	p	power	N	%	N	%	χ^2^	p	power
**Male sex**	52	39.7	32	46.4	0.83	0.36		27	42.9	15	40.5	0.05	0.82	
**Married**	33	25.2	18	26.1	0.02	0.89		27	42.9	14	37.8	0.24	0.62	
**Inpatient**	56	42.7	25	36.2	0.80	0.37		27	42.9	19	51.4	0.68	0.41	
**Urban residence**	58	44.3	29	42.0	0.09	0.76		32	50.8	19	51.4	<0.01	0.98	
**Diagnosis**					0.30	0.59	0.09					-0.43	0.52	0.10
**Schizophrenia**	96	73.3	53	76.8				48	76.2	26	70.3			
**Mood disorders**	35	26.7	16	23.2				15	23.8	11	29.7			
**Low education (Junior middle school or below)**	44	33.6	28	40.6	0.96	0.33		53	84.1	34	91.9	-	0.32	
**High monthly income (over RMB 1000)**	102	77.9	52	75.4	0.16	0.69		27	42.9	6	16.2	7.48	<0.01	
** Having insurance**	104	79.4	55	79.7	0.01	0.96		4	6.3	1	2.7	-	0.65	
**Patients’ stigma**														
**Awareness of mental disorder**	106	80.9	54	78.3	0.20	0.66	0.07	51	81.0	21	56.8	6.77	<0.01	
**Perception of being laughed at**	32	24.4	24	34.8	2.40	0.12	0.33	27	42.9	18	48.6	0.32	0.57	0.09
**Perception of being avoided**	27	20.6	24	34.8	4,78	<0.01		28	44.4	13	35.1	0.84	0.36	0.16
**Perception of being labeled as ‘mad’**	48	36.6	31	44.9	1.30	0.25	0.20	33	52.4	22	59.5	0.47	0.49	0.11
**Perception of being disinherited due to mental illness**	15	11.5	8	11.6	<0.01	0.98	0.05	4	6.3	7	18.9	3.76	0.05	0.49
**Perception of breakup due to mental illness**	20	15.3	23	33.3	8.74	<0.01		11	17.5	13	35.1	3.99	<0.05	
**Perception of family being embarrassed because of mental illness**	24	18.3	19	27.5	2.27	0.13	0.31	25	39.7	9	24.3	2.45	0.12	0.38
**Perception of loss of job due to mental illness**	39	29.8	29	42.0	3.03	0.08	0.40	29	46.0	19	51.4	0.26	0.61	0.08
**Perception of being denied promotions due to mental illness**	17	13.0	15	21.7	2.58	0.11		24	38.1	19	51.4	1.67	0.20	0.25
**Families’ stigma**														
**Awareness of mental disorder**	88	67.2	38	55.1	2.84	0.09	0.39	49	77.8	15	40.5	14.03	<0.01	
**Perception of being laughed**	34	26.0	27	39.1	3.70	>0.05	0.48	22	34.9	12	32.4	0.06	0.80	0.06
**Perception of being avoided**	31	23.7	26	37.7	4.36	<0.05	0.35	25	39.7	16	43.2	<0.01	0.95	0.06
**Perception of being labeled as ‘mad’**	35	26.7	20	29.0	0.12	0.73	0.06	31	49.2	23	62.2	1.58	0.21	0.24
**Perception of being disinherited due to mental illness**	4	3.1	10	14.5	8.06	<0.01		5	7.9	4	10.8	0.24	0.63	0.09
**Perception of breakup due to mental illness**	20	15.3	23	33.3	8.74	<0.01		11	17.5	13	35.1	3.99	<0.05	
**Perception of family being embarrassed because of mental illness**	26	19.8	18	26.1	1.03	0.31	0.17	22	34.9	14	37.8	0.09	0.77	0.38
**Perception of loss of job due to mental illness**	39	29.8	29	42.0	3.03	0.08	0.40	29	46.0	19	51.4	0.26	0.61	0.08
**Perception of being denied promotions due to mental illness**	23	17.6	16	23.2	0.91	0.34	0.15	20	31.7	17	45.9	2.02	0.16	0.29

^a^ DUP = duration of untreated psychosis.

Table 5 displays factors independently associated with long DUP for Chinese in binary logistic regression analyses after controlling for age. Compared to the short DUP group, patients having long DUP had more perception of breakup due to mental illness (OR = 2.22, 95% CI = 1.04–4.72, p = 0.04) and more families’ perception of patient being disinherited due to mental illness (OR = 6.47, 95% CI = 1.46–18.08, p = 0.01).

**Table 5:** Factors independently associated with long and short DUP in Changsha: binary logistic regression analysis^a^.

Variables	B	S.E.	Wald	p	Odds ratio	95% C.I.	power
**Constant**	-2.16	0.56	14.86	<0.01	0.12	-	
**Patients’ stigma**							
**Perception of being avoided**	0.60	0.37	2.64	0.10	1.82	0.88–3.76	0.95
**Perception of breakup due to mental illness**	0.80	0.39	4.25	0.04	2.22	1.04–4.72	0.99
**Families’ stigma**							
**Perception of being avoided**	0.39	0.36	1.15	0.28	1.47	0.73–2.97	0.67
**Perception of being disinherited due to mental illness**	1.64	0.64	6.47	0.01	5.13	1.46–18.08	1.00

^a^ Study site has been controlled as a covariate; Family’s perception of breakup due to mental illness has a collinearity with patients’ perception of breakup due to mental illness, therefore was not entered as an independent variable.

Table 6 displays factors independently associated with long DUP for Mauritians in binary logistic regression analyses after controlling for age. Compared to the short DUP group, patients with a DUP of > 3 months were almost less likely to have high monthly income (OR = 0.14, 95% CI = 0.04–0.51, p<0.01), while they had less patients’ awareness of mental illness (OR = 0.31, 95% CI = 0.11–0.92, p<0.05) and less families’ awareness of mental illness (OR = 0.14, 95% CI = 0.05–0.43, p<0.01).

**Table 6:** Factors independently associated with long and short DUP in Mauritius: binary logistic regression analysis^a^.

Variables	B	S.E.	Wald	p	Odds ratio	95% C.I.	power
**Constant**	2.02	1.00	4.09	0.04	7.55		
**High monthly income (over RMB 1000)**	-1.94	0.65	8.98	<0.01	0.14	0.04–0.51	1.00
**Patients’ stigma**							
**Awareness of mental disorder**	-1.17	0.55	4.50	<0.05	0.31	0.11–0.92	0.98
**Perception of breakup due to mental illness**	0.94	0.58	2.62	0.11	2.56	0.82–7.99	0.91
**Families’ stigma**							
**Awareness of mental disorder**	-1.94	0.56	12.21	<0.01	0.14	0.05–0.43	1.00

^a^ Study site has been controlled as a covariate; Family’s perception of breakup due to mental illness has a collinearity with patients’ perception of breakup due to mental illness, therefore was not entered as an independent variable.

## Discussion

**Fig 1:**
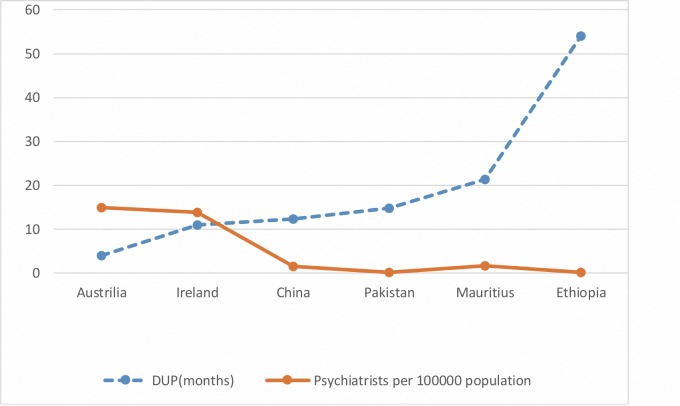
Comparison of months of mean DUP with the number of psychiatrists/100 000 population in Australia, Ireland, China, Pakistan, Mauritius and Ethiopia.

The mean DUP observed in Changsha and Mauritius from this study differ substantively from the same measurement in other countries. Developed countries like Australia [2–3] and Ireland [18, 29] have shown a relatively shorter DUP compared to our findings (2–6 months and 11 months respectively). The DUP measured in China is comparable to that of Pakistan (12.3 months as opposed to 14.8 months) [9] and the DUP measured in Mauritius (21.4 months) has been found to be longer than that of China and Pakistan but less than that of Ethiopia (54 months) [14]. These observations (Fig 1) show a gradual increase of mean DUP from Europe to Asian countries and even longer in African countries.

The relatively low DUP in developed countries like Australia and Ireland could be attributable to the fact that these countries health systems are likely to comprise of better infrastructure and have an efficient and developed referral system. Furthermore, such countries have more psychiatrists per 100 000 population as follows: 15.0 for Australia[30], and 13.9 for Ireland [30], as compared to 0.2 in Pakistan [31], 1.5 in China [31], 1.62 in Mauritius [32] and 0.14 in Ethiopia [33] which are depicted in Fig 1. The fact that developing countries have less psychiatrists per unit population would involve longer duration of transport to psychiatric consultation, and associated costs, which could have prevented these patients from approaching a psychiatrist for their mental health problems earlier. Moreover, it is more likely that such developed countries offer better insurance coverage for the treatment of mental illness. Additionally, costs incurred for consultation with a psychiatrist, with the associated costs of medication seem to be more of an economic burden in developing countries as compared to developed countries [34]. Another reason as to why such developed countries may have a shorter DUP could be due to increased mental health literacy [35] which could be due to mental health education programs and anti-stigma campaigns. Community support is also likely to be present in such countries [36], thereby encouraging the mentally ill to seek early psychiatric help.

Moreover, lay people from developed countries seem to attribute the cause of psychotic illness to the biopsychosocial model as opposed to non-Western cultures which tend to attribute the cause of mental illness to supernatural phenomena like witchcraft and possession by evil spirits [37]. For instance in Pakistan, Malaysia and Ethiopia, a large proportion of patients suffering from psychotic illnesses preferred consulting alternative methods of treatment instead of a psychiatrist for treatment because of such beliefs [14, 30,38]. This study mirrors such findings in China and Mauritius as well. These factors could have subsequently led to delay in psychiatric help in such countries.

In this study, no diagnosis of mental illness was an independent correlate of a long DUP in both sites. Research focused on people with first episode psychosis were not consistent in average length of DUP in schizophrenia and mood disorders. For instance, large sample studies have found mean DUP ranging from 8 to 48 weeks in schizophrenia [2, 39], while it tended to be longer in bipolar disorder, ranging from 5 to 10 years [40–42]. However, in a study comprised of 375 participants, Renwick et al. [43] found the mean DUP was the shortest in major depressive disorder (8.7±14.6 months), followed by bipolar disorder (17.3±40.5 months), and longest for those with schizophrenia (25.8±30.4 months). Due to the small proportion of mood disorders in our samples, as well as to the lack of following up those with major depressive disorder, we can’t draw the conclusion that a difference of DUP exists between affective and non-affective psychoses, towards which further research should be aimed.

We surprisingly found correlation between long DUP and a low monthly income in Mauritius, but not in Changsha. In Changsha, medical insurance covers both urban and rural people, who can receive a large proportion of reimbursement over medical expenses. In Mauritius, the whole health system is totally free of all charge, which means that despite free consultations, treatment, inpatient stay and medications, a low monthly income contributed to a long DUP. This could maybe imply that the low monthly income was associated with other factors, for instance, beliefs in other causes of psychosis and being unaware of being mentally ill by this lower socioeconomic group. This could maybe imply that just improving the economic level can strengthen people’s awareness of the recognition and timely treatment of mental illness in Mauritius, but not in Changsha.

The impact of stigma in these two developing regions also presented diversity. In Changsha, the patient’s perception that conjugal relationships could be broken up due to the presence of mental illness was independently associated with a long DUP. This shows that, for fear of breakup of relationships, patients with mental illness, despite knowing that they suffer, do not approach a psychiatrist early for fear of breakup. Moreover, a significant proportion of family members seem to have made the patient late for psychiatric consultation because they feared that the patient would be disinherited on grounds of mental illness. This shows that help-seeking behavior on the part of the patient is influenced at the core level of the family and associated relationships. In Mauritius, decreased awareness of mental illness and related issues from patients and the family members was an independent correlate associated with a long DUP. This makes sense, given the fact that being unaware that the patient is ill will, of course, lead to delay in seeking psychiatric help. Although no significant difference was found in frequency of long DUP between Changsha and Mauritius, this study suggests that cultural and societal contexts, including the individual and the family, contribute to determinants of DUP, and racial or ethnic identity might have different interactions with both barriers and pathways to mental health services.

Our study has some limitations. The studied population in the current study is heterogeneous and small, which might limit the generalizability of the findings. We calculated post hoc power of all the comparison with no significant differences (except for general demographic variables) when settingαerr prob = 0.05. And the value ranged from 0.07 to 0.67. The highest required sample size should be more than 10 000. So maybe some negative results are also potential influencing factors to a long DUP, which need further study with larger sample size. In addition, much data was collected from self-reports, and has therefore been affected by a social desirability bias and recall bias, particularly in cases where some questions were regarded as privacy matters. Despite these limitations, this study has provided new information in many ways. First, it studies two totally different and diverse cultures across two countries of different continents and compares not only their DUP, but also various treatment barriers like geographic and economic barriers, stigma, alternative beliefs and approaches, amongst others. Second, it identifies the problem of help-seeking and DUP in Mauritius, a research that has never been done before in this country. Third, this study has also assessed different perceptions of different members of the same family concerning the patient’s mental illness, which has revealed differences in opinion concerning psychiatric treatment approach. This shows a disturbance at the level of the family, especially family relations, concerning treatment approach at the onset of psychosis. Fourth, this study also reveals new information concerning DUP in affective and non-affective psychoses. However, one of the limitations is that we only have a relatively small sample size. Moreover, the study in both sites was hospital based. Therefore the findings should be carefully extrapolated to that of the general population. More accurate findings would have been obtained had the sampling been done at the community level.

## Conclusion

In conclusion, this study has identified the following factors to be independent correlates of DUP: the perception of the patient that mental illness can cause the breakup of his/her couple and perception of the family member that mental illness can cause the patient to be disinherited for Chinese in Changsha, and a low monthly income and decreased patient and family members’ awareness of mental illness for the Mauritian patient. This shows that in both Changsha and Mauritius, mental health education campaigns have to be initiated so as to increase mental health literacy, and the target groups could be family members, especially spouses/partners of patients, and the general public. The lower socio-economic class should be paid more attention to in Mauritius. The findings of this study also give us basic data on which to guide further research in this field at the community level. It also gives food for thought concerning DUP in patients and families with racial, ethnic and cultural differences. Further research should aim to understand the cause of difference of DUP in specific social and cultural backgrounds or economic conditions with the hope that findings of such research can help identify factors which may predispose to a long DUP and thus devise ways to help reduce DUP to a minimum.

## Supporting Information

S1 File Relevant raw data underlying the findings described in manuscript. (ZIP)
